# Research progress of ankyrin repeat domain 1 protein: an updated review

**DOI:** 10.1186/s11658-024-00647-w

**Published:** 2024-10-17

**Authors:** Xusan Xu, Xiaoxia Wang, Yu Li, Riling Chen, Houlang Wen, Yajun Wang, Guoda Ma

**Affiliations:** 1 Maternal and Child Research Institute, Shunde Women and Children Hospital, Guangdong Medical University, Foshan, 528300 China; 2 Department of Neurology, Longjiang Hospital, Foshan, 528300 China; 3 Department of Pediatrics, Shunde Women and Children Hospital, Guangdong Medical University, Foshan, 528300 China; 4 Medical Genetics Laboratory, Shunde Women and Children Hospital, Guangdong Medical University, Foshan, 528300 China; 5 Respiratory Research Institute, Shunde Women and Children Hospital, Guangdong Medical University, Foshan, 528300 China

**Keywords:** Ankrd1, Cardiovascular diseases, Skeletal muscle diseases, Angiogenesis, Tumor therapy

## Abstract

Ankyrin repeat domain 1 (Ankrd1) is an acute response protein that belongs to the muscle ankyrin repeat protein (MARP) family. Accumulating evidence has revealed that Ankrd1 plays a crucial role in a wide range of biological processes and diseases. This review consolidates current knowledge on Ankrd1’s functions in myocardium and skeletal muscle development, neurogenesis, cancer, bone formation, angiogenesis, wound healing, fibrosis, apoptosis, inflammation, and infection. The comprehensive profile of Ankrd1 in cardiovascular diseases, myopathy, and its potential as a candidate prognostic and diagnostic biomarker are also discussed. In the future, more studies of Ankrd1 are warranted to clarify its role in diseases and assess its potential as a therapeutic target.

## Introduction

The muscle ankyrin repeat protein (MARP) family, comprising Ankrd1, Ankrd2 (also known as ARPP), and Ankrd23 (also referred to as DARP), is a conserved group characterized by shared structural motifs including the coiled-coil domain, nuclear localization signal (NLS), and tandem ankyrin repeats (ANK) [1]. Ankrd1 is further distinguished by the presence of PEST motifs associated with protein degradation and a putative nuclear export signal (NES) [2]. Initially discovered in human endothelial cells [3], Ankrd1 has garnered significant attention due to its abundant expression in myocardial tissue and its heightened sensitivity to the chemotherapeutic agents adriamycin/doxorubicin, earning it the names cardiac ankyrin repeat protein and cardiac adriamycin-responsive protein (CARP) [4].

Historically, research on Ankrd1 predominantly addressed its implications in cardiomyopathies [5–7]. However, recent years have seen a broadening of focus, with studies exploring its involvement in diverse conditions such as cancers [8], muscular diseases [9], and viral infections [10]. Concurrently, there has been a surge in identifying upstream regulators and interacting proteins that influence Ankrd1’s function. This review aims to provide a comprehensive overview of Ankrd1’s roles in health and disease, highlighting recent advancements and identifying gaps in current knowledge that suggest promising directions for future research.

## The regulation and modification of Ankrd1

**Fig. 1:**
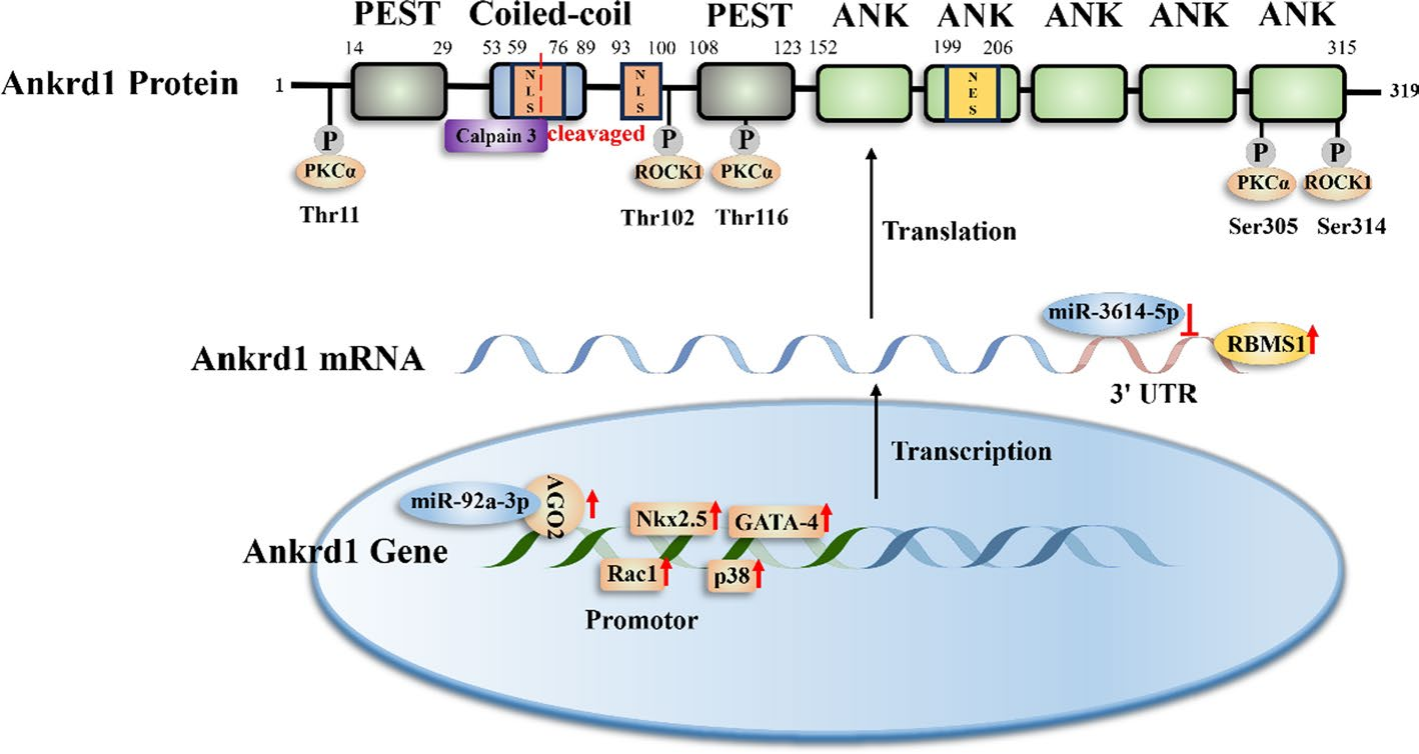
Schematic representation of Ankrd1 structure and molecular regulation of Ankrd1 at different levels. At the transcriptional level, p38, Rac1, and transcription factors Nkx2.5 and GATA-4 bind to the Ankrd1 promoter and positively regulates Ankrd1 expression. The miR-92a-3p can translocate into the nucleus and positively mediate Ankrd1 expression by AGO2. At the posttranscriptional level, miR-3614-5p binds to the 3′UTR of the Ankrd1 gene and decrease Ankrd1 expression. The RNA-binding protein RBMS1 can also bind to the 3′ untranslated region (UTR) and increase the mRNA stabilization of Ankrd1. The Ankrd1 protein has 319 amino acids, including one coiled-coil domain, two PEST-like regions, two putative nuclear localization signals (NLSs), five tandem ankyrin repeats (ANK), and one potential nuclear export signal (NES). At the posttranslational level, Ankrd1 can be phosphorylated by PKCα and ROCK1. Calpain 3 can cleavage by Ankrd1 protein at the first NLS. This diagram is not drawn to scale

As a rapid-response protein, the expression of Ankrd1 is regulated across various biological layers, including transcription, post-transcription, translation, and post-translation (Fig. 1). This multifaceted control system allows Ankrd1 to swiftly adapt to changing cellular environments and mediate appropriate responses.

Transcriptional regulation: the transcription factor NK2 homeobox 5 (Nkx2.5) and GATA binding protein 4 (GATA-4) are pivotal in this regulatory landscape. These factors bind cooperatively to the Ankrd1 promoter in rat cardiomyocytes, significantly enhancing gene expression [5]. The involvement of MAPK14/p38 and Rac1, which interact with the M-CAT element within the promoter, also upregulated the transcription of Ankrd1 [11]. Such multifactorial control underscores the precision with which Ankrd1 expression is modulated in response to cellular cues.

Epigenetic modulation also plays a critical role in the regulation of Ankrd1. Previous studies found high level of DNA methylation of Ankrd1 in several human cancer cell lines, such as A427, LNCaP, MCF7, MeWo, and BxPC-3 cells [12, 13]. However, our recent analysis based on the TCGA database showed decreased DNA methylation in the promoter region of Ankrd1 in patients with breast cancer, lung cancer, or pancreatic cancer [14]. This bidirectional modulation by DNA methylation highlights the gene’s responsive adaptation to diverse oncogenic environments.

Further, Ankrd1 is identified as a downstream target of the YAP/TAZ signaling pathway, a revelation that enriches our understanding of its regulatory complexity [15–17]. This integration into broader signaling networks illustrates how Ankrd1 is situated at the nexus of multiple regulatory axes, enabling a highly responsive transcriptional response to stress in varying cellular contexts.

Post-transcriptional regulation: microRNAs (miRNAs) are central to the post-transcriptional regulation, with several miRNAs identified as modulators of Ankrd1 expression. For instance, miR-3614-5p notably suppresses Ankrd1 expression in osteosarcoma cells by directly targeting the 3′ untranslated region (3′-UTR) of its mRNA, illustrating the specific post-transcriptional silencing mechanisms employed by cells [8]. Additionally, predictive analyses from resources like the GSCALite and ENCORI databases suggest potential targeting by miR-10a-5p, miR-10b-5p, miR-28-3p, and miR-425-5p, though these interactions await experimental validation [14]. Further complexity is added by miR-92a-3p, which enhances Ankrd1 expression through an unusual mechanism involving its translocation into the nucleus, where it positively influences transcription [18].

The maintenance of mRNA stability is a key point for many biological processes. Ankrd1 mRNA contain instability element AU-rich motifs suggesting that Ankrd1 may also be regulated at the level of mRNA stability [3]. TGF-β could enhance the mRNA stability of Ankrd1 in C2/2 cells [19]. A recent study reported that Ankrd1 levels rise significantly in the early stages of fibroblast senescence induced by etoposide. This increase is attributed to robust mRNA stabilization facilitated by the RNA-binding protein RBMS1 [20].

Translational and post-translational modifications: post-translational modifications (PTMs) such as phosphorylation play pivotal roles in modulating the structural configuration and biological activity of Ankrd1. Notably, protein kinase C (PKCα) targets Ankrd1 at specific residues—Thr11, Thr116, and Ser305—potentially altering its structural conformation and functional capabilities [21]. Similarly, Rho-associated coiled-coil containing protein kinase 1 (ROCK1) modifies Ankrd1 at Thr102 and Ser314, which are crucial for its subcellular localization and protein-binding ability, thereby impacting its role in cellular signaling pathways [22].

Beyond phosphorylation, the stability and degradation of Ankrd1 are regulated by the ubiquitin–proteasome pathway, highlighting the protein’s turnover dynamics. The 26S proteasome plays a central role in this process, where the PEST motifs within Ankrd1 facilitate its recognition and subsequent degradation, ensuring timely cellular responses to stress [23, 24]. Furthermore, Ankrd1 is a substrate for the calcium-dependent cysteine protease Calpain 3, which adds another layer of complexity to its post-translational regulation, influencing both its half-life and functional state in response to calcium signaling [25].

The comprehensive regulation of Ankrd1 at these various levels underscores its importance as a highly responsive protein within cellular stress responses. Each regulatory layer ensures that Ankrd1 can quickly adapt to changing cellular environments, which is critical for its role in modulating apoptosis and other cellular functions. Further research into these regulatory mechanisms will enhance our understanding of Ankrd1’s roles in health and disease, potentially leading to novel therapeutic approaches for diseases where Ankrd1’s activity is dysregulated.

## The role of Ankrd1 in myocardial system

**Fig. 2:**
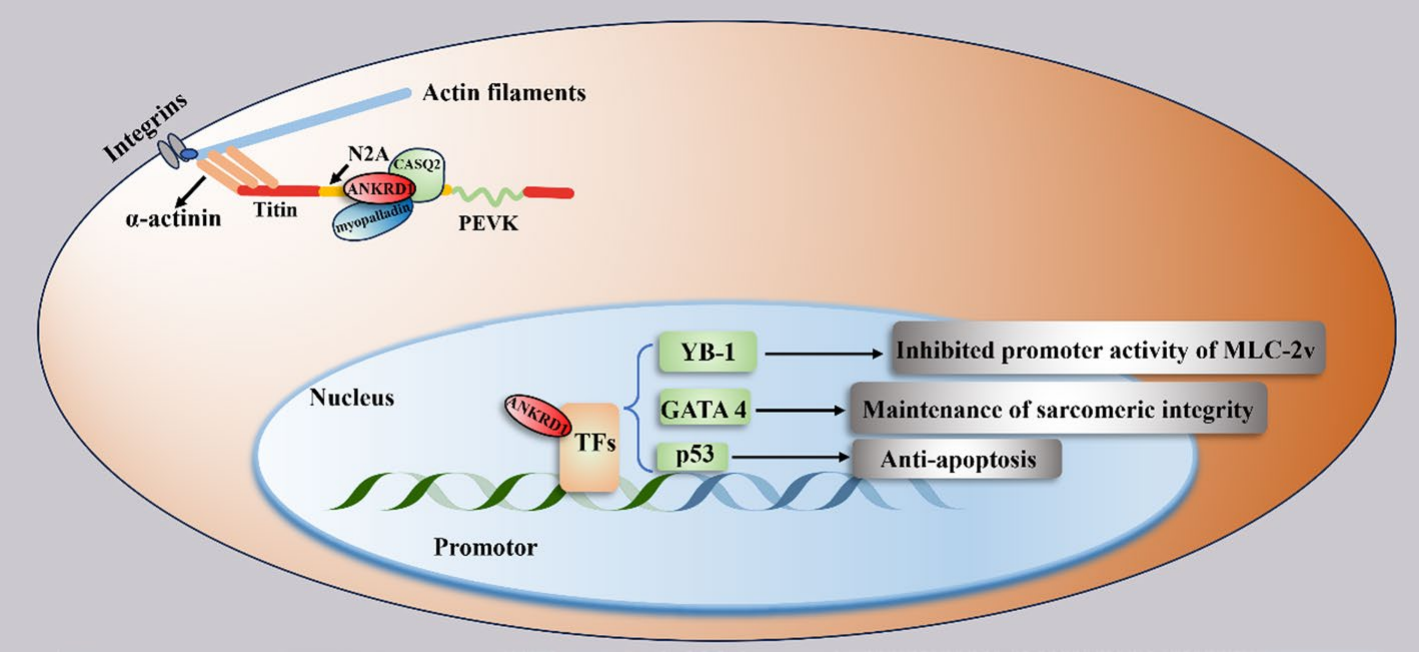
The cellular roles of Ankrd1 in cardiomyocytes. As a transcriptional regulator, Ankrd1 participates in the regulation of transcriptional activity by interacting with different transcriptional factors (TFs), including YB-1, GATA 4, and p53. The biological functions in cells regulated by Ankrd1 and these TFs are displayed in gray boxes. Ankrd1 is also a structural protein, and contributes to the structural integrity of the sarcomere by interacting with titin, CASQ2, and myopalladin. This figure was improved on the basis of the study by Samaras SE et al. [27]

Ankrd1 exhibits a dual nuclear-cytoplasmic localization in myocardium, playing distinct roles in each compartment [26]. In the nucleus, it acts as a transcriptional regulator of gene expression by interacting with various transcription factors such as YB-1, GATA 4, and p53, thereby influencing a range of transcriptional activities [4, 27]. In the cytoplasm, Ankrd1 functions as a structural protein, contributing to the integrity of the sarcomere by interacting with key muscle proteins such as titin and myopalladin [27, 28]. Figure 2 provides a comprehensive illustration of Ankrd1’s cellular roles in striated muscle cell, highlighting both its regulatory function in the nucleus and its structural role in the sarcomere. Additionally, the figure outlines the biological processes regulated by Ankrd1 in coordination with these proteins, with specific functions displayed in grey boxes. This dual role of Ankrd1, dependent on its localization, underscores its critical contribution to both transcriptional regulation and the mechanical stability of muscle cells, reflecting its importance in both physiological and pathological contexts [29].

### Ankrd1 in cardiac development and regeneration

Ankrd1 is predominantly expressed in the heart, particularly in cardiomyocytes rather than cardiac fibroblasts, underscoring its critical role in myocardial function [4]. Ankrd1 is categorized among the fetal genes, akin to atrial natriuretic peptide (ANP) and BNP [5]. Even before the formation of heart tube, as early as 7.5 days post-coitum, Ankrd1 mRNA is detectable in the embryonic cardiogenic plate, suggesting its early involvement in heart development [4]. As the heart’s chambers take shape, the distribution of Ankrd1 mRNA shows a subtle predilection for the atrium over the ventricle, with levels peaking in the atrium and outflow tract regions such as the pulmonary artery and ascending aorta by embryonic days 10.0 to 10.5. Post these stages, although the expression diminishes, it remains discernible and gradually decreases throughout the developmental timeline into postnatal stages [4, 30]. Moreover, ectopic expression of Myopalladin’s NH2-terminal, which competes with endogenous Ankrd1, disrupts the sarcomeric integrity of cardiac myocytes [31].

Functional studies reinforce the importance of Ankrd1 in cardiogenesis. For instance, enhanced Ankrd1 expression has been linked to heart regeneration capabilities, observed through comprehensive transcriptomic and proteomic analyses in relevant mouse models [32]. Furthermore, Ankrd1’s interaction with Y-box binding protein 1 (YB-1) leads to the suppression of myosin light chain-2 (MLC-2v), a ventricular chamber-specific gene, signifying its regulatory capacity during heart tube formation [30]. This process is intricately coordinated with crucial transcription factors Nkx2.5 and GATA-4, which are critical for the heart tube formation and ventral morphogenesis [5], highlighting a complex regulatory network essential for early cardiac architecture. More direct evidence comes from Snezana’s team, who discovered this in a zebrafish cardiac injury model. They found that Ankrd1 is specifically upregulated in cardiomyocytes surrounding the injury site following cardiac ventricle cryoinjury. This response highlights its involvement in the early stages of heart repair and adaptation to stress [33]. These insights collectively depict Ankrd1 as a mediator in the complex orchestration of cardiac development and regeneration.

### Ankrd1 and cardiovascular diseases

Research has demonstrated Ankrd1’s association with several cardiovascular conditions, including hypertrophic cardiomyopathy (HCM), dilated cardiomyopathy (DCM), ischemic cardiomyopathy (ICM) [34], and drug-induced cardiomyopathy [35].

#### Hypertrophic cardiomyopathy

Ankrd1 plays a crucial role in the pathophysiology of HCM by regulating gene expression linked to cardiac muscle function and structural integrity and interacting with key cardiac signaling pathways that control heart muscle growth and stress responses. The Ankrd1’s upregulation has been reported in various cardiac hypertrophy models in vitro and in vivo, including those induced by transverse aortic constriction (TAC), hypertensive stimuli, and hormonal influences like isoprenaline and angiotensin II [5, 11, 36–38].

As a classical component of the Hippo pathway, Ankrd1 contributes to the regulation of organ size, underscoring its broader role in organ development and disease pathogenesis [39, 40]. Notably, Ankrd1 levels correlate with the expression of brain natriuretic peptide (BNP), a key antihypertrophic marker in cardiovascular disease models [11]. However, studies like those by Bang, M. L. et al. reveal that the absence of Ankrd1 does not manifest typical cardiac phenotypes under normal or stress conditions like TAC, suggesting that its role may be compensated by other mechanisms or context-dependent [41]. In-depth studies have shown that the overexpression of Ankrd1 initially enhances atrial contractility, which paradoxically diminishes over time, hinting at a complex regulatory mechanism influenced by the duration and context of expression [42]. These observations are supported by the varied cardiac responses observed in in vitro and in vivo hypertrophy models, where Ankrd1 upregulation was consistently reported [5, 11, 36, 37] (Table 1).

**Table 1:** Intervention of Ankrd1 in cardiomyocytes

Diseases	Stimulus	Cell lines/animals	Intervention of Ankrd1	Functional effects	Association with disease	Ref.
Physiology	–	H9c2 cells	Overexpression with plasmid	Nonphosphorylatable form of Ankrd1 repressed the promoter activity of MLC-2v	Nonphosphorylatable form of Ankrd1 decreased the cell size of H9c2 cells more efficiently than wild-type Ankrd1	[22]
Physiology	–	H9c2 cells	Overexpression with plasmid	Increased the protein level of p53; decreased the protein level of Mdm2	–	[59]
Physiology	–	H9c2 cells	Overexpression with plasmid	Increased the expression of hypertrophic proteins p-ERK1/2 and p-GATA4	–	[48]
Physiology	–	C57BL/B6 mice	Global deletion of MARP	Normal cardiac morphology and cardiac function; did not change the heart/body weight ratios	–	[41]
Physiology	–	Male mice	Global deletion of Ankrd1	Normal cardiac function	–	[28]
Physiology	–	C57/BL6J mice	Cardiac-specific overexpressing	No physiological abnormalities	–	[47]
Physiology	–	Mice	Cardiac-specific overexpressing	Cardiomyocyte disorganization and myofibrillar disruption	Developed sinus venosus defect and progressive ventricular diastolic dysfunction	[42]
AIC	Doxorubicin	Adult rat ventricular myocytes	Knockdown with siRNA	Inhibited myofilament gene transcription and disrupted cardiomyocyte sarcomere structure; reversed the GATA4 rescue of the doxorubicin-induced sarcomere phenotype	–	[7]
AIC	Doxorubicin	Adult rat ventricular myocytes	Overexpression with adenovirus	Unable to rescue the doxorubicin-induced sarcomere disarray phenotype	–	[7]
DCM	MLP knockout	Sv129/black swiss mice	Global deletion of Ankrd1	Prevented the formation of Ankrd1/PKCα/PLCβ1 complex, and inhibited the accumulation of PKCα at the intercalated discs	Alleviated the DCM phenotype in MLP knockout mice	[29]
DCM	EAM	BALB/c mice	Global deletion of Ankrd1	Mitigated myocarditis-induced cardiac damage/remodeling	–	[50]
HCM	Ang II	NRVCs	Overexpression with adenovirus	Upregulated the expression of ANP, β-MHC and calcineurin	Enhanced Ang II-induced myocyte hypertrophy	[38]
HCM	Ang II	Neonatal rat cardiomyocytes	Overexpression with adenovirus	Enhanced the mitochondrial translocation of Bax and phosphorylated p53, increased mitochondrial permeability and cardiomyocyte apoptosis, and reduced cell viability	–	[60]
HCM	Ang II	Neonatal rat cardiomyocytes	Knockdown with shRNA	Decreased the mitochondrial translocation of Bax and phosphorylated p53, inhibited mitochondrial permeability and cardiomyocyte apoptosis, and increased cell viability	–	[60]
HCM	Isoprenaline	Engineered heart tissue	Overexpression with adenovirus	Did not change the basal force of contraction; decreased the contractile response to Ca^+^ and isoprenaline in engineered heart tissue	–	[37]
HCM	Isoprenaline	C57/BL6J mice	Cardiac-specific overexpressing	Decreased the myocyte area and fibrosis; decreased the left ventricular posterior wall thickness, HW/BW and HW/TL ratios	Inhibited isoprenaline-induced cardiomyocyte hypertrophy	[47]
HCM	Phenylephrine	Neonatal rat cardiomyocytes	Overexpression with adenovirus	Decreased the myocyte area; reduced the expression of the hypertrophic molecular markers α-actin, β-MHC, and ANF; inhibited the MAPK/ERK signaling pathway	Inhibited phenylephrine-induced hypertrophy	[47]
HCM	Phenylephrine	NRVCs	Knockdown with siRNA	Decreased phenylephrine-induced phosphorylation of ERK1/2 and GATA4, inhibited nuclear translocation of the Ankrd1 complex	Inhibited phenylephrine-induced hypertrophy	[28]
HCM	Phenylephrine	Male mice	Global deletion of Ankrd1	–	Inhibited phenylephrine-induced hypertrophy	[28]
HCM	TAC	C57BL/6 male mice	Overexpression with adenovirus	Increased the cytosolic CARP level, the heart weight/body weight ratio, and the expression of calcineurin	–	[38]
HCM	TAC	C57BL/6 male mice	Overexpression with adenovirus	Increased the lung weight/body weight ratio, decreased the left ventricular fractional shortening, increased cardiomyocyte apoptosis and the expression of phosphorylated p53	–	[60]
HCM	TAC	C57BL/6 male mice	Overexpression of nuclear Ankrd1	Induced cardiac remodeling by activating MYH7	–	[18]
HCM	TAC	C57/BL6J mice	Cardiac-specific overexpressing	Decreased the myocyte area and fibrosis; inhibited the decrease in α-MHC and SERCA2 expression induced by TAC; decreased the left ventricular posterior wall thickness and the LV mass; no difference in cardiac function (EF%, FS%); inhibited the MAPK/ERK and TGF-β/Smad3 signaling pathway	Inhibited TAC-induced cardiomyocyte hypertrophy	[47]
HCM	TAC	C57BL/6 male mice	Knockdown with shRNA	–	Inhibited TAC-induced hypertrophy	[38]
HCM	TAC	C57BL/6 male mice	Knockdown with shRNA	Inhibited TAC-induced cardiomyocyte apoptosis and the expression of phosphorylated p53	–	[60]
HCM	TAC	Male mice	Global deletion of Ankrd1	–	Did not affect TAC-induced hypertrophy	[28]
HCM	TAC	C57BL/B6 mice	Global deletion of MARP	–	Did not affect TAC-induced hypertrophy	[41]
ICM	Hypoxia	H9c2 cells	Overexpression with plasmid	Diminished the hypoxia-induced apoptosis	–	[61]
ICM	H/R	Neonatal mouse ventricular cardiomyocytes	Overexpression with adenovirus	Overexpressed Ankrd1 was mainly distributed in the nucleus; increased Bcl-2 gene expression	Decreased H/R-induced apoptosis	[62]
ICM	H/R	Neonatal mouse ventricular cardiomyocytes	Knockdown with shRNA	–	Enhanced H/R-induced apoptosis	[62]

HCM, hypertrophic cardiomyopathy; DCM, dilated cardiomyopathy; ICM, ischemic cardiomyopathy; Ang II, angiotensin II; NRVCs, neonatal rat ventricular cardiomyocytes; ANP, atrial natriuretic peptide; β-MHC, β-myosin heavy chain gene; TAC, transverse aortic constriction; shRNA, short hairpin RNA; MARP, muscle ankyrin repeat protein; EF%, ejection fraction percentage; FS%, fractional shortening percentage; AIC, adriamycin-induced cardiomyopathy; H/R, hypoxia/reoxygenation

**Fig. 3:**
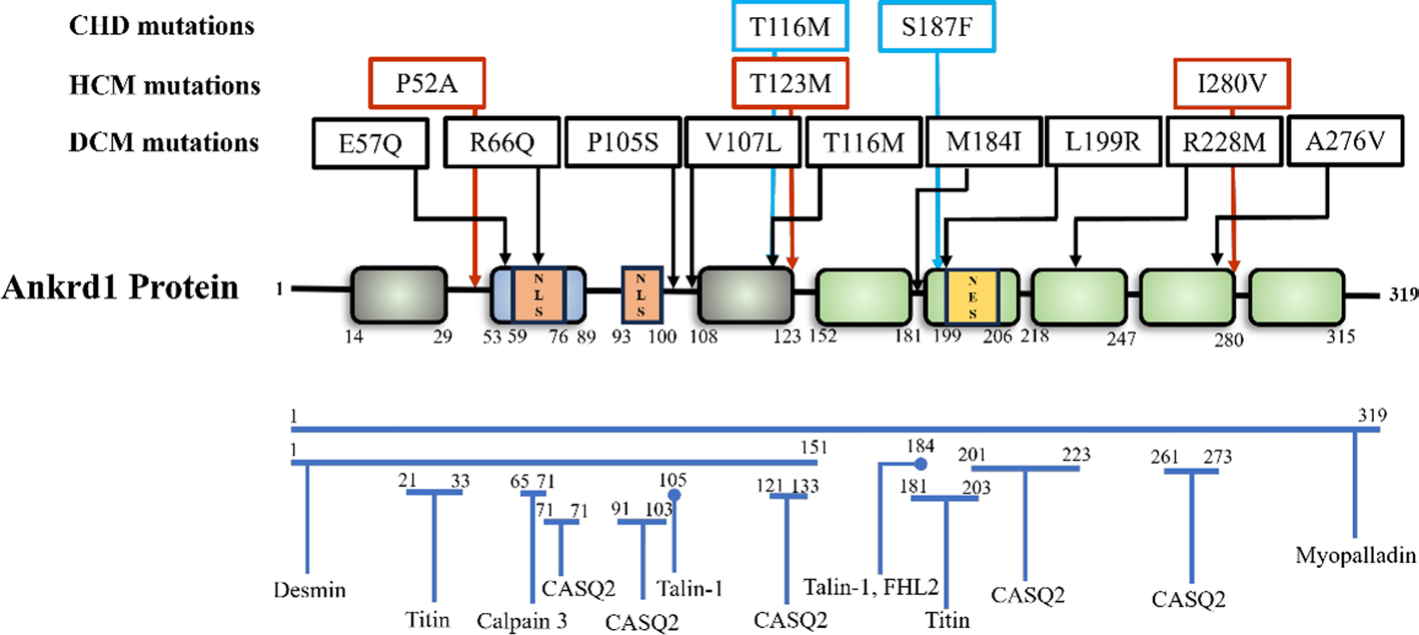
Genetic studies have identified several Ankrd1 mutations in CHD, HCM and DCM. The middle part of the figure is a schematic representation of human Ankrd1 structure. The upper part of the figure shows the specific location of mutations in protein. The lower part of the figure shows the interaction protein of Ankrd1 with known specific binding sites. This figure was improved on the basis of the study by Ling SSM et al. [2]

Genetic studies have identified specific Ankrd1 mutations associated with HCM (Fig. 3, Table 2). In a 384 HCM cohort, three missense mutations (P52A, T123M, and I280V) within Ankrd1 were found [43]. Later functional studies found that all of them increased the binding of Ankrd1 to both titin and myopalladin [43]. These mutations impact Ankrd1's stability and localization, affecting its function at the sarcomeric Z–I bands and its regulatory capacity in the nucleus [43, 44]. For instance, while wild-type Ankrd1 modulated contraction responses negatively in engineered heart tissues, the T123M mutation exhibited a heightened contractile response [37, 44].

**Table 2:** Ankrd1 mutations identified in the patient cohort

Diseases	Identified mutations^a^	Functions	Ref.
HCM	P52A	Increased its binding to titin and myopalladin; displayed higher intensity at the Z–I bands and diffused localization in the cytoplasm, nuclear, and/or at nuclear membrane in the mature cardiomyocytes	[43]
DCM	E57Q	Decreased the repressor activity of Ankrd1 on MLC2v promoter; reversed the inhibition of Ankrd1 on phenylephrine-induced myocardial hypertrophy	[52]
DCM	R66Q	–	[52]
DCM	P105S	Lost its binding to talin-1; enhanced the down-regulation of p53 and upregulation of myogenin	[51]
DCM	V107L	Blocked the decreased expression of TGF-β1 and CASQ2; enhanced the down-regulation of EGR1 and decreased the expression of TNNT1	[51]
DCM	T116M	–	[52]
CHD	T116M	Increased the stability of the Ankrd1 protein; enhanced its repressive effect on ANF promoter activity	[54]
HCM	T123M	Increased its binding to titin and myopalladin; displayed higher intensity at the Z–I bands and diffused localization in cytoplasm, nuclear, and/or at nuclear membrane in the mature cardiomyocytes; Ankrd1T123M-transduced engineered heart tissues (EHTs) showed higher contraction parameters	[43, 44]
DCM	M184I	Lost its binding to talin-1 and FHL2	[51]
CHD	S187F	Enhanced its repressive effect on ANF promoter activity; decreased the nuclear distribution of Ankrd1	[53]
DCM	L199R	Decreased the repressor activity of Ankrd1 on MLC2v promoter; reversed the inhibition of Ankrd1 on phenylephrine-induced myocardial hypertrophy	[52]
DCM	R228M	–	[63]
DCM	A276V	Decreased the repressor activity of Ankrd1 on MLC2v promoter; reversed the inhibition of Ankrd1 on phenylephrine-induced myocardial hypertrophy	[52]
HCM	I280V	Increased its binding to titin and myopalladin; displayed higher intensity at the Z–I bands and diffused localization in cytoplasm, nuclear and/or at nuclear membrane in the mature cardiomyocytes	[43]

HCM, hypertrophic cardiomyopathy; DCM, dilated cardiomyopathy; CHD, congenital heart defect; NLS, nuclear localization signal; ANK, tandem ankyrin repeats; PEST, protein degradation sequences motifs; FHL2, 4-and-a-half LIM domains 2; TGF-β1, transforming growth factor β1; CASQ2, calsequestrin 2; EGR1, early growth response factor; TNNT1, troponin T1; ANF, atrial natriuretic peptide

^a^Amino acid change caused by mutations identified in Ankrd1

Although rare variants within Ankrd1 identified in cardiomyopathy cohorts have been supported by functional effects in cellular models. However, large scale human genetic studies of cardiomyopathy found that Ankrd1 did not have a significant overall excess of variants in cases [45, 46]. Walsh et al. found no significant enrichment of rare Ankrd1 variants in HCM cases compared to controls, suggesting that it is unlikely to play a major causative role in HCM [46]. In addition, in a reassessment involving 7855 cardiomyopathy patients and 60,706 reference samples, the team also reported that many previously identified rare variants in Ankrd1 do not exhibit the pathogenic significance initially attributed to them, further reducing its clinical relevance in cardiomyopathy [45]. Consequently, studying the effect of variants in Ankrd1 on clinical phenotype in large family pedigrees or patient cohorts may help to clarify the relationship between Ankrd1 variation and cardiomyopathy.

Further research underscores the complexity of Ankrd1's role in cardiac hypertrophy, including its interaction with the calcineurin-NFAT signaling pathway and the MAPK/ERK and TGF-β/Smad3 pathways, which are crucial in mediating hypertrophic responses [38, 47]. The therapeutic potential of targeting Ankrd1 is highlighted by interventions like Baoyuan decoction (BYD), which ameliorates hypertrophy through the Ankrd1-ERK/GATA4 pathway, suggesting a nuanced but significant impact on cardiac health [48].

#### Dilated cardiomyopathy

DCM is characterized by ventricular dilation and impaired cardiac contractility. Elevated expression levels of Ankrd1 have been observed in patients with DCM, particularly in those with end-stage heart failure, where Ankrd1 expression and protein levels were notably higher [34, 49]. Importantly, the levels of Ankrd1 negatively correlated with measures of cardiac function, including contractility and left ventricular compliance [49].

In experimental models, heart-specific overexpression of Ankrd1 in mice demonstrated a decline in atrial contractility by 10 months of age, suggesting a deleterious role of elevated Ankrd1 in cardiac function [42]. Additionally, myocarditis, a common precursor to DCM, has been linked to changes in Ankrd1 expression. Mice models deficient in Ankrd1 expression showed reduced cardiac remodeling and preserved contractility when challenged with myocarditis-induced DCM [50].

Genetic studies have identified several missense mutations within Ankrd1 associated with DCM (Fig. 3, Table 2). Moulik et al. identified mutations such as P105S, V107L, and M184I within a cohort of 208 DCM patients. Notably, the M184I mutation was found to disrupt the binding of Ankrd1 to Talin 1 and FHL2, critical components of the sarcomere, while the P105S mutation affected binding to Talin 1, highlighting the importance of these interactions in maintaining sarcomeric integrity [51]. Another study by Duboscq-Bidot et al. identified additional mutations like T116M, A276V, E57Q, R66Q, and L199R, with some such as A276V, E57Q, and L199R reducing the repressive activity of Ankrd1 on the MLC2v promoter, which plays a role in cardiac muscle contraction [52].

Moreover, Ankrd1’s interaction with signaling molecules such as PKCα, which is upregulated in DCM, suggests a complex regulatory network. In failing hearts with end-stage DCM, Ankrd1 modulates the localization of PKCα and PLCβ1, preventing their relocation to the intercalated discs, a process linked to the progression of DCM. The global genetic ablation of Ankrd1 led to normal PKCα phosphorylation and a lack of signaling at the intercalated discs, which was associated with a reduced progression of DCM symptoms [29]. Overall, though the above evidence suggests that Ankrd1 may contribute to the progress of DCM, further studies are necessary to fully elucidate the mechanisms by which Ankrd1 contributes to the development and progression of DCM.

#### Congenital heart defects (CHDs)

CHDs represent the most prevalent type of birth defects, with cardiac septal defects comprising over half of these cases. Research has identified a heterozygous missense variant (S187F) within the Ankrd1 gene in a family with a history of cardiac septal defects [53]. This variant, situated in a highly conserved region of Ankrd1, notably enhances the protein’s repressive effect on the ANP promoter activity and adversely affects the nuclear localization of Ankrd1, suggesting a potential mechanistic link to septal defect pathology [53]. Total anomalous pulmonary venous return (TAPVR), another severe congenital heart defect characterized by the abnormal connection of pulmonary veins to the right atrium or its branches, has been associated with a mutation (T116M) in Ankrd1. This mutation, located in the PEST motif of the protein, appears to increase Ankrd1’s stability and its transcriptional repression activity on the ANP promoter, implicating it in the etiology of TAPVR in several sporadic cases [54].

Recent experimental models have provided further insights into Ankrd1’s involvement in congenital heart defects. Mice with heart-specific overexpression of Ankrd1 have been shown to develop sinus venosus defects, adding to the evidence of Ankrd1’s critical role in cardiac development [42]. These findings highlight the need for ongoing research into the genetic underpinnings of CHDs, specifically how Ankrd1 mutations influence cardiac morphology and function, potentially offering new avenues for early diagnosis and targeted therapies in congenital heart disorders.

#### Anthracycline-induced cardiotoxicity

Anthracycline antibiotics (ANTs), such as daunorubicin (DAU) and doxorubicin (Dox), are widely recognized for their efficacy against a range of tumors but are limited in clinical use due to their high incidence of cardiotoxicity [55]. This cardiotoxicity necessitates the development of reliable biomarkers for early detection and management. In this context, Ankrd1 has emerged as a potential biomarker, particularly in pediatric acute lymphoblastic leukemia (ALL).

In heart diseases, such as heart failure, serum Ankrd1 can be used as a diagnostic marker, but these studies were conducted in adults [56]. To this end, we explored the possibility of Ankrd1 as a cardiac injury in children with ALL. Our team recently found that, after DAU chemotherapy in acute lymphoblastic leukemia (ALL), serum Ankrd1 concentration decreased in patients with adverse cardiac events (ACE), but increased in patients without ACE, and the extent of Ankrd1 concentration changes was negatively correlated with the grade of ACE. Further analysis indicates that serum Ankrd1 concentration is an effective predictor of ANT-induced acute ACE, with more sensitivity and specificity than QT interval corrected (QTc), creatine kinase MB (CK-MB), and traditional biomarkers high-sensitivity troponin T (TNT-HS) [57]. The relevance of Ankrd1 extends beyond just a biomarker for cardiotoxicity. Its serum levels also correlate with the QT interval, which is associated with calcium metabolism and excitation–contraction coupling, highlighting its potential role in broader cardiac physiological processes [56, 57]. This association further underscores the importance of Ankrd1 in cardiac health and disease management. Mechanistically, Dox has been shown to suppress Ankrd1 expression by inhibiting its promoter activity, leading to sarcomere disarray in cardiac tissues. This disruption is not mitigated by overexpression of Ankrd1, indicating that while Ankrd1 reduction is a marker of damage, its increase alone does not confer protection against Dox-induced damage [7, 35]. However, overexpression of GATA4 has been shown to modestly restore Ankrd1 expression and partially ameliorate Dox-induced sarcomere disarray, suggesting a pathway for potential therapeutic intervention [7].

These findings have drawn significant attention, with commentary from specialists in oncocardiology lauding the clinical translational value of this research, particularly its implications for improving therapeutic outcomes in pediatric oncology patients [58]. Further in vivo studies are recommended to deepen understanding of the mechanisms by which Ankrd1 influences cardiotoxic responses and to validate therapeutic strategies aimed at mitigating anthracycline-induced cardiac damage.

#### Atherosclerosis

Ankrd1 plays a multifaceted role in atherosclerosis, acting both as a modulator of inflammatory responses and as a regulator of cell proliferation within the vascular wall. A previous study demonstrated the specific localization of Ankrd1 in endothelial cells and intimal smooth muscle cells within human atherosclerotic plaques, indicating its role in these vascular components; notably, Ankrd1 was absent in the medial SMCs and lesion-associated macrophages [64]. In models of mouse atherogenesis, Ankrd1 expression was shown to be dynamic, increasing in activated SMCs and disappearing when SMCs were in a static state [64].

The expression of Ankrd1 is modulated by several cytokines and growth factors implicated in atherosclerosis. Activin A, known as an inhibitor of atherogenic processes, was found to enhance Ankrd1 expression in SMCs, suggesting a protective role of Ankrd1 in atherosclerosis [64]. Inflammatory cytokines such as IL-1 and TNF-α, along with TGF-β—a critical mediator of vascular remodeling and atherosclerosis—have been identified as potent inducers of Ankrd1 expression [9, 19].

The anti-inflammatory properties of Ankrd1 were further illustrated by studies showing that Ankrd1 could exert feedback inhibition on NF-κB transcriptional activity, a key pathway in inflammatory signaling [65]. Additionally, Ankrd1 has been implicated in the negative regulation of SMC proliferation, a fundamental process in plaque development. Kanai et al. reported that Ankrd1 could inhibit the pro-proliferative effects of TGF-β on SMCs by inducing the expression of the cyclin-dependent kinase inhibitor p21 and inhibiting the phosphorylation of the retinoblastoma protein (Rb), which are critical regulators of the cell cycle [19]. These findings suggest that the balance of Ankrd1 activity may influence the progression of atherosclerosis, offering potential therapeutic targets for managing or preventing this widespread and impactful cardiovascular disease.

#### Ischemic cardiomyopathy and arrhythmogenic right ventricular cardiomyopathy

Ankrd1 mRNA expression is significantly elevated in ICM, suggesting its involvement in the pathological processes associated with this condition [34, 66, 67]. Conversely, Ankrd1 expression is notably reduced during ischemia–reperfusion (IR) injury in the myocardium, indicating a differential role depending on the cardiac stress and injury context [68]. Additionally, Ankrd1 has been identified as a biomarker in the infarct boundary zones, highlighting its potential utility in defining the extent of myocardial infarction [69].

Further illustrating the complex role of Ankrd1, studies in cardiac cells reveal divergent outcomes based on different conditions. For instance, overexpression of Ankrd1 in H9c2 cells under hypoxic conditions reduces apoptosis, suggesting a protective role in myocardial cells [61]. In contrast, in neonatal rat cardiomyocytes, Ankrd1 exacerbates apoptosis when exposed to angiotensin-II, indicating a condition-dependent pro-apoptotic function in the heart [60]. Moreover, in myocardial ischemia/reperfusion models, overexpression of Ankrd1 downregulates p53 expression, activates anti-apoptotic Bcl-2, and attenuates apoptosis, revealing its complex interaction with the p53 signaling pathway in cardiac health [62].

In the context of traditional medicine, Storax, widely used in Chinese medicinal practices, has been shown to protect cardiomyocytes against myocardial fibrosis and cardiac dysfunction. This protective effect is mediated through inhibition of the AT1R-Ankrd1-p53 signaling pathway in a rat model of acute myocardial infarction induced by isoproterenol hydrochloride (ISO) [70]. Such findings underscore the therapeutic potential of targeting Ankrd1-related pathways in cardiac treatment strategies.

Further emphasizing the role of Ankrd1 in cardiac diseases, its expression is also upregulated in desmin-related cardiomyopathy, where Ankrd1 interacts with the type III intermediate filament desmin. This interaction is regulated through the Akt-NF-κb signaling pathway in human airway smooth muscle cells (HASMCs), suggesting a broader regulatory role in muscle cell function [66, 71, 72].

Ankrd1’s involvement extends to arrhythmogenic right ventricular cardiomyopathy (ARVC), where abnormal increases in Ankrd1 expression have been observed [73]. Additionally, a genetic study identified a nonsense variant (Q529X) in myopalladin, which disrupts Ankrd1 localization and sarcomeric Z-disc integrity, contributing to fibrotic remodeling in restrictive cardiomyopathy (RCM) [74, 75]. This mutation further implicates Ankrd1 in the structural and functional integrity of cardiac muscle.

These diverse findings across various forms of cardiomyopathy highlight the complex role of Ankrd1 in cardiac pathophysiology. The protein’s involvement in different signaling pathways and its response to various cardiac injuries make it a potential target for therapeutic interventions aimed at mitigating the progression of cardiomyopathic conditions.

## The role of Ankrd1 in muscular system

### Ankrd1 in muscle development and regeneration

In skeletal muscle development, the expression of Ankrd1 is evident during the early stages of muscle morphogenesis, being first detectable around embryonic day 12.5 and disappearing in most muscles by day 17.5 after the definitive muscle pattern is established [9]. This pattern is mirrored during muscle regeneration, such as in the response to bupivacaine-induced injury, where Ankrd1 is also induced early in the regenerating muscle [76]. Interestingly, the upregulation of Ankrd1 in muscle regeneration is delayed in obese mice, paralleling the delayed expression of traditional muscle regeneration markers in these animals [77].

The role of Ankrd1 extends beyond development to muscle maintenance, where it is vital for the structural integrity of muscle fibers. Knockdown of Ankrd1 disrupts the formation of myotubes and reduces the expression of key muscular proteins including myosin heavy chain (MHC), myoD, and caveolin-3 [78]. This disruption is also evident in the structural disarray of myofibrils, emphasizing the importance of Ankrd1 in maintaining sarcomeric organization [7].

Furthermore, Ankrd1 is integral to the cellular architecture of striated muscle cells, particularly within the I-band of the sarcomere, where it is known to increase rapidly under mechanical stress conditions such as passive stretch, eccentric contractions, or unilateral diaphragm denervation [26, 79, 80]. The structural integrity of the sarcomere, maintained by passive force largely generated by titin filaments, is crucial, and Ankrd1 influences this by modulating the passive tension and stiffness of myofibrils. Notably, incubation with recombinant Ankrd1 results in increased passive tension [80, 81]. However, other studies found that only knocking out Ankrd1 along with the other two MARP family members can lead to decreased fiber stiffness and an extended resting sarcomere length due to the expression of a longer isoform of titin [41, 82].

Unlike that in mammals, in poultry-specific expression of Ankrd1 in skeletal muscles has been linked to the regulation of muscle organ size, acting as a downstream target gene of muscle growth inhibitor-myostatin. Studies in various species including humans have shown that loss of function mutations in Myostatin lead to a double-muscled phenotype. In chicken myoblasts, Ankrd1 promotes cell proliferation and inhibits apoptosis, suggesting its involvement in biological evolution and the regulatory mechanisms of Myostatin [26].

At the molecular level, Ankrd1 binds specifically to the UN2A and Ig81 segments of titin’s N2A domain, increasing the unfolding force of Ig81 and masking the phosphorylation site of the titin-N2A region, crucial for the strain- and phosphorylation-dependent signaling in skeletal muscle [81, 83]. Moreover, it forms a complex with titin-N2A and F-actin in C2C12 cells and heart lysates, which is sensitive to Ca^2+^ levels, indicating its role in mechano-transduction [80]. The interaction with the thin filament components, such as tropomyosin, troponin, and actin, ensures that Ankrd1-mediated passive force enhancement is contingent upon the integrity of these filaments, showcasing the sophisticated regulatory mechanisms Ankrd1 exerts in muscle function and response to mechanical stress.

### Ankrd1 in muscle pathophysiology

Ankrd1 plays multifaceted roles in muscle physiology, intricately interacting with molecular pathways that govern muscle atrophy, regeneration, and inflammation. It indirectly interacts with muscle atrophy-related genes such as NF-κB p50 and Bcl-3, signaling its involvement in muscle catabolic processes [84]. Ankrd1 has a molecular structure similar to that of IκB, the inhibitor of NF-kB. Consequently, overexpression of Ankrd1 diminishes NF-κB p65 DNA-binding activity and inhibits TNF-α-induced NF-κB transcriptional activity, thereby potentially moderating inflammatory responses in muscle tissues [25, 65]. Paradoxically, Ankrd1 also associates with muscle-specific RING finger ubiquitin ligases MuRF1 and MuRF2, which are key mediators of muscle atrophy, illustrating the complex regulatory roles Ankrd1 may have in muscle protein turnover [85]. Additionally, Ankrd1 interacts with the androgen receptor, reversing the transcriptional inhibition of muscle-specific ubiquitin E3 ligases such as muscle atrophy F-box (MAFbx) in mouse C2C12 myoblasts [86]. It also enhances mRNA levels of genes associated with myogenesis, such as myogenin, MEF2, p21, and troponin I1 in L6 myoblasts [86].

In response to various stressors, changes in muscle cell size and myofibrillar remodeling are observed, with Ankrd1 expression being upregulated in conditions such as androgen deprivation therapy [87], chronic obstructive pulmonary disease [88], or senescence [89], indicating its role in the adaptive responses of striated muscle cells to chronic stresses [92–94]. Conversely, treatments such as nandrolone that counteract denervation atrophy also increase Ankrd1 expression, suggesting a protective or compensatory role in such contexts [90].

Further underscoring its clinical relevance, Ankrd1 expression is prominently upregulated in skeletal muscles affected by a range of myopathies including motor neuron diseases such as amyotrophic lateral sclerosis (ALS), spinal muscular atrophy (SMA), and Kennedy disease, as well as various muscular dystrophies such as Duchenne and Fukuyama congenital muscular dystrophy [76, 91–93]. Its expression correlates with disease severity, particularly noted in the selective expression in severely atrophic myofibers of ALS patients and during muscle regeneration phases [91, 92]. Intriguingly, Ankrd1 expression declines upon successful reinnervation in transient denervation scenarios, and exhibits a negative correlation with lifespan in SOD1^G93A^ ALS mouse models [94], while positively correlating with creatine kinase activity in skeletal myopathies induced by peroxisome proliferator-activated receptors [95].

These findings highlight Ankrd1 as a potentially sensitive biomarker for muscle atrophy and disease progression. However, despite extensive research, the detailed mechanisms through which Ankrd1 influences muscle health—spanning regeneration, remodeling, inflammation, and atrophy—are yet to be fully elucidated.

## Ankrd1 in cancer

**Fig. 4:**
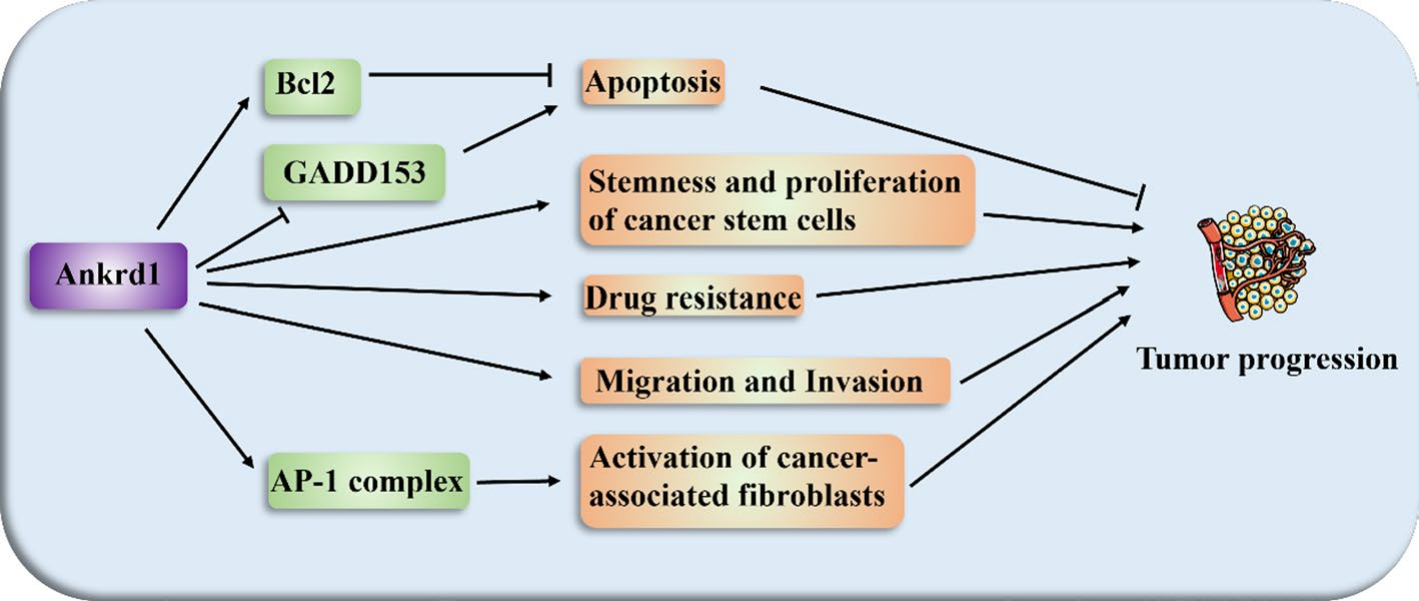
Ankrd1 participates in tumor progression. Ankrd1 inhibits apoptosis by Bcl2 and GADD153. Ankrd1 promotes stemness and proliferation of breast cancer stem cells. Ankrd1 enhances drug resistance, such as cisplatin, phenylbutyrate, afatinib, and osimertinib. Ankrd1 promotes tumor migration and invasiveness by inhibiting apoptosis in colorectal cancer cell lines. Ankrd1 activates the pro-tumorigenic properties of cancer-associated fibroblasts via AP-1 complex

Ankrd1 has emerged as a significant player in various cancer types, influencing tumor progression and therapeutic resistance through distinct molecular pathways (Fig. 4). Its expression is significantly upregulated in various cancers, which underscores its role in cancer biology. Here, we delve deeper into the specific functions and implications of Ankrd1 in different cancers, illustrating its pervasive influence on disease outcomes.

Ovarian cancer: in ovarian cancer, the overexpression of Ankrd1 significantly impacts the disease’s pathophysiology and treatment outcomes by modulating endoplasmic reticulum (ER) stress-related apoptosis pathways. Ankrd1 affects the expression of crucial apoptotic regulators such as GADD153 and Bcl2. By upregulating Bcl2, a well-known anti-apoptotic protein, and decreasing the levels of GADD153, which can have complex roles in apoptosis regulation, Ankrd1 promotes tumor survival. This modulation not only enhances the survival of cancer cells but also confers resistance to chemotherapeutic agents such as cisplatin. The result is a notably reduced sensitivity to apoptosis in ovarian cancer cells, thereby complicating the efficacy of standard treatments and potentially leading to poorer patient outcomes [96, 97].

Lung cancer: similarly, in lung cancer, Ankrd1 corelated with the resistance to targeted therapies. Its expression is associated with decreased effectiveness of tyrosine kinase inhibitors like afatinib and osimertinib, which are designed to target EGFR-mutant non-small cell lung cancer (NSCLC). Ankrd1 enhances cell survival through its anti-apoptotic functions, thereby allowing cancer cells to evade the cytotoxic effects of these drugs. The presence of Ankrd1 thus represents a significant challenge in the management of lung cancer, as it impairs the response to therapies that are otherwise effective in targeting specific oncogenic drivers in this disease [98].

Colorectal cancer: Ankrd1 displayed increased expression in intestinal adenomas and was induced in mouse models of Wnt/β-catenin-induced tumors [99]. The activation of the Wnt/β-catenin signaling pathway is a key mechanism involved in the development and progression of intestinal adenomas. Increased expression of Ankrd1 has been linked to poor prognosis of colorectal cancer [14]. By inhibiting apoptosis, Ankrd1 contributes directly to the tumorigenic process, promoting the metastasis and invasion of colorectal cancer cells [14].

Osteosarcoma: in osteosarcoma (OSA), Ankrd1 is targeted by the tumor-suppressing miR-3614-5p, and its expression is regulated by the interaction with RGMB-AS1, a long non-coding RNA. Overexpression of Ankrd1 or suppression of miR-3614-5p counteracts the effects of RGMB-AS1 silencing, promoting tumor proliferation and invasion. Thus, Ankrd1 plays a critical role in OSA by modulating cancer cell dynamics and represents a potential therapeutic target [8].

Breast cancer: in breast cancer, Ankrd1 not only mediates resistance to drugs like phenylbutyrate but also plays a critical role in the epithelial-mesenchymal transition (EMT). Its expression, induced by EMT transcription factors such as SNAI1, enhances the stemness and proliferative capacity of breast cancer stem cells, underscoring its complex role in cancer biology [100, 101]. In triple-negative breast cancer cells, the induction of Ankrd1 expression by the autophagy inducer and mTORC1 inhibitor rapamycin suggests variable roles in different cell lines [102].

Given its pivotal role in fostering tumor progression and conferring drug resistance, targeting Ankrd1 presents a compelling strategy for enhancing cancer treatment. By inhibiting Ankrd1, it may be possible to obstruct crucial oncogenic pathways, thereby improving patient prognoses and augmenting the efficacy of chemotherapeutic agents. Specifically, the disruption of Ankrd1 interactions within the microenvironment of tumors, such as in cancer-associated fibroblasts, has demonstrated significant potential. For example, in skin cancer models, interfering with Ankrd1 signaling in these fibroblasts has effectively reversed their tumor-promoting characteristics, suggesting a potential for broader application in other cancer types [103].

While the research on Ankrd1’s involvement in cancer progression and resistance to therapy offers promising insights, several critical limitations remain that must be addressed to fully leverage its potential as a therapeutic target. Firstly, much of the existing evidence relies heavily on cellular models and bioinformatics predictions, which, while informative, do not entirely replicate the complex interactions and dynamics of living organisms. This reliance highlights a significant gap in our understanding that can only be bridged by increasing the number of in vivo animal studies, which will provide a more realistic assessment of Ankrd1’s biological functions and therapeutic potential in a systemic context. Additionally, the current body of research lacks substantial clinical trials that include diverse ethnic populations. This deficiency is critical because genetic variations across different populations can significantly influence both the progression of diseases and responses to therapy. Therefore, to truly ascertain the viability of targeting Ankrd1 in cancer treatment, it is essential to conduct more comprehensive in vivo studies and to initiate large-scale clinical trials that encompass a broad demographic spectrum.

## The role of Ankrd1 in wound healing, angiogenesis, and fibrosis

**Fig. 5:**
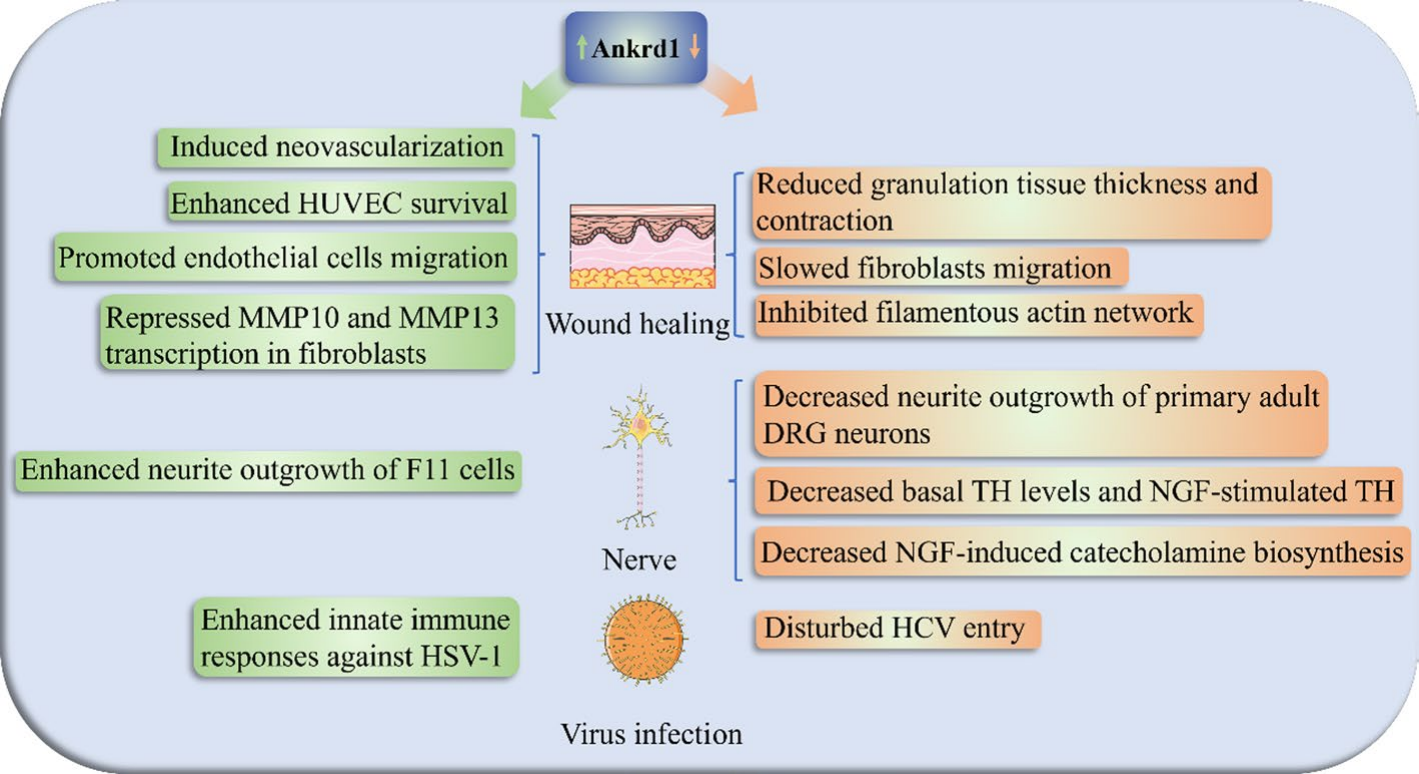
Ankrd1 plays important roles in wound healing, nerves, and virus infection. The green text box shows the effects after knock-down or knock-out Ankrd1 expression, the yellow text box represents the performance after Ankrd1 overexpression

Ankrd1’s mRNA and protein expression were increased in the excisional wound (Fig. 5) [104]. After skin wounding, Ankrd1 protein is expressed sequentially in inflammatory cells, and endothelial cells, and the subsequent expression pattern is similar to that of intact skin, expressed in the panniculus carnosus and hair follicles [105]. Global deletion of Ankrd1 (Ankrd1^−/−^) delayed wound healing, manifested as reduced granulation tissue thickness, and decreased contraction [105]. These clues suggested a crucial role of Ankrd1 in wound healing, and the effects of Ankrd1 in various types of cells are jointly involved in this process.

Endothelial cells participate in tissue repair from multiple perspectives, including metabolic reprogramming, receptor trafficking and endothelial to mesenchymal transition (EMT) [106]. Ankrd1 expression was increased in injured arteries [19]. Occlusion of the femoral artery, an arteriogenesis model, also found increased Ankrd1 expression [107]. Another study reported that vascular endothelial growth factor (VEGF), a key driver for neovascularization [108], also enhanced the Ankrd1 expression [109]. Overexpression of Ankrd1 induced neovascularization, increased blood perfusion in excisional wound model and ischemic wounds, enhanced human umbilical vein endothelial cells (HUVEC) survival and promoted endothelial cells migration [104]. Overexpression of Ankrd1 in cos-1 cells increased early growth response 1 (Egr-1) expression [107]. The hypothesis that Ankrd1 could promote angiogenesis via the Egr-1/FGF2 pathway [110] is an intriguing subject for future study. These findings suggested a potential role of Ankrd1 in neovascularization, and the specific mechanism needs to be further explored.

Fibroblasts are highly dynamic cells that play a central role in tissue repair [111]. Fibroblasts isolated from Ankrd1^−/−^ mice migrated slowly on collagen or fibronectin matrix and have poor filamentous actin network [105]. Mechanistically, Ankrd1 represses the activator protein 1 (AP-1)-dependent transactivation of matrix metallopeptidase 10 (MMP10) and MMP13 in fibroblasts [112]. Increased Ankrd1 expression induced fibroblast activation through interacting with AP-1 family proteins (c-JUN and c-FOS), and then binding to the promoter and enhancer regions of fibroblast effector genes (Acta2, Has2, and Col1a1) [103].

Excessive formation of matrix deposition caused by pathological tissue repair will lead to fibrosis. Ankrd1 has been mentioned in several organ fibrosis, including cardiac fibrosis [113], hepatic fibrosis [16], and renal fibrosis [114]. The activated hepatic stellate cells (HSCs) isolated from CCl4-induced and common bile duct ligation (CBDL)-induced liver fibrosis model increased Ankrd1 expression, which was completely dependent on YAP [16]. Ankrd1 was proven as one of YAP1 target genes [39], which is crucial for coordinating fibrogenesis after organ injury [115]. The mTORC2 pathway is another important signaling pathway for promoting organ fibrosis [114]. The disruption of the mTORC2 or Yap/Taz pathway attenuated the fibrosis progress and decreased the Ankrd1 expression [114]. Another study reported that TGF-β1-induced and unilateral ureter obstructive (UUO)-induced renal fibrosis also upregulated Ankrd1 expression [114]. Ankrd1 is a direct downstream target of TGF-β/Smad signaling [19], which was activated in most fibrotic disorders [115]. A recent study showed moderate fibrosis, enhanced myofibril loss, and increased TGFβ expression and ERK1/2 phosphorylation in ventricular of heart-specific Ankrd1 overexpression mice [42].

In summary, Ankrd1 is important for wound healing, promotes angiogenesis, and is implicated in fibrosis. It facilitates wound repair and tissue regeneration, enhances endothelial functions for improved blood vessel formation. These roles highlight Ankrd1 as a potential therapeutic target in conditions involving abnormal healing and fibrosis. Further studies on Ankrd1 could lead to targeted therapies that better manage these processes.

## Ankrd1 in osteogenic differentiation and osteoporosis

Ankrd1 has emerged as a regulator in the osteogenic differentiation of various stem cell populations. Ankrd1 was induced along with enhanced osteogenesis in bone morphogenetic protein 2 (BMP2)-transfected adipose stem cells (ASCs), which implies a potential role of Ankrd1 in osteogenesis [109]. The increased Ankrd1 expression was also found in human mesenchymal stem cells (hMSCs) [116] and mice bone marrow-derived mesenchymal stem cells (BMSCs) during osteogenic differentiation [117].

However, the function of Ankrd1 in osteoblastogenesis appears complex and context dependent. For instance, Yi et al. reported that knocking down Ankrd1 in hMSCs upregulated osteogenic markers, increased the activity of alkaline phosphatase (ALPL), and enhanced matrix mineralization, implying that Ankrd1 may act as an inhibitor of osteoblastogenesis [116]. Conversely, a later study found that increased Ankrd1 expression promoted osteogenic differentiation of BMSCs. This promotion does not extend to proliferation but is mediated through the activation of the ERK signaling pathway, Wnt signaling pathway, and the modulation of caveolin-3 (CAV3) ubiquitination [117]. Additionally, the impact of Ankrd1 extends to pathological conditions such as osteoporosis. Bilateral ovariectomy, a model for osteoporosis, leads to decreased expression of Ankrd1 in BMSCs. Interestingly, the ectopic expression of Ankrd1 in this model can alleviate some effects of osteoporosis induced by ovariectomy, which is linked to the regulatory effects of estrogen on osteoclast formation and bone resorptive activity [117, 118].

## Ankrd1 in neural regeneration and neurotransmitter synthesis

Ankrd1 also involves in the neural response to injury and the maintenance of neurotransmitter synthesis, displaying cell type-specific effects on neurogenesis. Notably, Ankrd1 expression is upregulated in dorsal root ganglion (DRG) neurons following peripheral sciatic nerve injury and is colocalized with growth-associated protein 43 (Gap43), a marker of Schwann cells [119]. This protein significantly enhances neurite outgrowth in F11 cells and primary adult DRG neurons, underscoring its potential in promoting neural regeneration [119]. Conversely, studies in PC12 pheochromocytoma cells indicate that Ankrd1 does not affect neurite outgrowth, suggesting that its regenerative effects may be specific to certain cell types [120].

Further elucidating its neural role, Ankrd1 is inducible by nerve growth factor (NGF) through extracellular signal-regulated kinase 5 (ERK5) pathways, but not ERK1/2, highlighting a specific signaling cascade that supports neuron survival and homeostasis in the central nervous system [120]. This regulation is crucial as NGF is essential for the maintenance of the sympathetic nervous system and mediates central nervous system neuron homeostasis through tyrosine kinase signal transduction [121]. Moreover, ERK5 itself is implicated in adult hippocampal neurogenesis [122]. Ankrd1 also influences catecholamine synthesis. The absence of Ankrd1 elevates the ubiquitination of tyrosine hydroxylase (TH), the key enzyme in catecholamine biosynthesis, leading to reduced levels of dopamine, norepinephrine, and epinephrine in PC12 cells [120]. Since these neurotransmitters are vital for the transmission of neural signals and overall nervous system functionality, Ankrd1’s role in their synthesis underpins its broader protective function in neural health.

However, the effects of Ankrd1 extend beyond the peripheral nervous system. Hypoxia, a significant factor in various neurological disorders, substantially suppresses Ankrd1 expression [61, 123]. Despite these advances in understanding Ankrd1's peripheral roles, its impact on the central nervous system remains largely unexplored.

## Ankrd1 in inflammation and viral infections

Ankrd1 was recognized as a cytokine-inducible gene in human dermal microvascular endothelial cells (HDMEC), where its mRNA expression is rapidly induced by inflammatory stimuli including interleukin-1a (IL-1α), TNF-α, and bacterial lipopolysaccharide (LPS) [3]. This induction suggests a significant role for Ankrd1 in inflammatory processes, potentially mediated through the NF-κB signaling pathway. Notably, Ankrd1 interacts with NF-κB p50 and modulates inflammatory responses in C2C12 cells by inhibiting NF-κB p65 activity, indicating a feedback loop that could regulate inflammation [65].

The role of Ankrd1 extends beyond simple inflammatory responses to being a critical player in the host–pathogen interaction during various viral infections. Its deregulation has been observed in several viral contexts, including COVID-19 [124], early infections by human papillomavirus (HPV) types 11, 16, and 45 [125], and hepatitis C virus (HCV) infection [126, 127]. Ankrd1 expression is upregulated in cells infected with HCV, driven by interactions with the nonstructural 5A (NS5A) protein. This interaction, occurring via the domain II of NS5A and the C-terminal region of Ankrd1, not only boosts Ankrd1 promoter activity but also influences intracellular calcium homeostasis and ER stress, contributing to HCV pathogenesis [127]. Additionally, silencing Ankrd1 impairs HCV propagation, underlining its necessity for viral entry and highlighting its potential as a target for therapeutic intervention [127]. Moreover, Ankrd1 has been implicated in human immunodeficiency virus (HIV) infection through its mediation of interactions between the HIV-1 envelope protein gp120 and the integrin receptor α4β7 on B cells, further emphasizing its role in the pathogenesis and immune response during viral infections [128].

Ankrd1 also involves in dermatological and viral interactions, particularly in patients with atopic dermatitis who suffer from eczema herpeticum following herpes simplex virus infection. In these cases, Ankrd1 mRNA levels are significantly decreased in peripheral blood mononuclear cells, indicating a compromised immune response [129]. Conversely, its overexpression has been shown to enhance innate immune defenses against HSV-1 by interacting with interferon regulatory factor 3 (IRF-3), thereby augmenting the production of type I and type III interferons, crucial elements in the antiviral response [10].

In conclusion, Ankrd1 is a key regulator in both inflammation and viral responses, influencing NF-κB activity and interacting with various viral proteins. Its dual role in managing inflammatory reactions and facilitating viral pathogenesis highlights its potential as a therapeutic target. Further elucidation of Ankrd1’s mechanisms could lead to improved strategies for modulating immune responses in disease contexts.

## Ankrd1 in renal disorders

The role of Ankrd1 extends to renal disorders. In a study by Keiko Matsuura et al., immunohistochemical analysis of 69 renal biopsy samples demonstrated differential Ankrd1 expression across glomerular diseases. Ankrd1 was notably present in crescentic glomerulonephritis, diabetic nephropathy, and lupus nephritis, yet absent in endocapillary glomerulonephritis, minimal change disease, and normal kidneys. Notably, Ankrd1 levels were higher in lupus nephritis cases with nephrotic syndrome compared to those without. Absent in membranous glomerulonephritis, but detectable in membranous lupus nephritis, Ankrd1’s distinct expression patterns serve both as a disease severity marker and a differential diagnostic tool for similar renal pathologies [130]. Additionally, in renal epithelial HK-2 cells, Ankrd1 is markedly upregulated in response to calcium oxalate exposure, which mimics the conditions of kidney stone formation. This significant differential expression of Ankrd1 exacerbates cell damage through activation of the p53/SLC7A11 axis, highlighting its critical role in the cellular response to kidney stone-related stress [131].

## Ankrd1 in reproductive system

In reproductive health, Ankrd1 shows impacting the complex intercellular interactions within the ovarian follicle, particularly through its involvement in inflammatory processes and its association with exosomal regulation. In the ovarian follicle, exosomes enhance the communication pathways between granulosa cells and the maturing oocyte, with Ankrd1 being identified as a highly expressed gene in granulosa cells. This suggests its importance in regulating the local inflammatory environment, potentially influencing the oocyte's developmental competence and readiness for fertilization [132]. Moreover, Ankrd1’s expression during critical phases of oocyte quality management underscores its role in influencing cellular mechanisms such as apoptosis and stress responses, essential for oocyte maturation. The interplay of Ankrd1 with other elements like cadherins, integrins, and the extracellular matrix adds to the follicle's structural and functional integrity [133].

## Summary and perspectives

In this review, we summarize the present understanding of Ankrd1 function, revealing the current role of Ankrd1 in myocardium and skeletal muscle development, neurogenesis, bone formation, angiogenesis, wound healing, fibrosis, apoptosis, inflammation, and infection. We also provided a review of the current findings of Ankrd1 in cardiovascular diseases and myopathy as well as its potential as a biomarker.

Several important issues need to be addressed regarding Ankrd1. Early studies have found that Ankrd1 can express in both cytoplasm and nucleus and undergo intracellular translocation to respond to different stimuli [38]. Y. Lei et al. further showed that Ankrd1 can colocalize with mitochondria or ER in ovarian cancer cells [97]. This begs several questions: Can Ankrd1 also locate mitochondria or ER in other types of cells? How does it achieve intracellular translocation? What is the function of this intracellular translocation of Ankrd1? Then, phosphorylation modification has displayed its modulation on the function and intracellular localization of Ankrd1 [22]. However, little is currently known about the post-translational modifications of Ankrd1, and no research reported the levels of phosphorylated or other post-translational modified Ankrd1 in diseases. In addition, only a small number of downstream targets and interacting proteins of Ankrd1 have been identified. Previous studies have predicted abundant downstream targets and interacting proteins of Ankrd1 through two yeast hybridization, chromatin immunoprecipitation (ChIP), and commercial transcription factor interaction assay [22, 103, 112, 134]. Utilizing these resources or further genetic screening will help to better understand the potential role of Ankrd1 in diseases.

Ankrd1 has been proposed to have potential as a prognostic and diagnostic biomarker for cardiomyopathy [57], RMS [135], SMA [92], and other diseases [91]. However, the limited patient cohorts and short follow-up periods reduce the robustness of these findings. To validate Ankrd1’s utility as a clinical biomarker, extensive multicenter randomized controlled trials with adequate design are essential. Further investigation into Ankrd1’s functions promises deeper insights into its disease mechanisms, potentially unveiling novel therapeutic avenues in clinical settings.

## Data Availability

No data was used for the research described in the article.

## Abbreviations

Ankrd1: Ankyrin repeat domain 1
MARP: Muscle ankyrin repeat protein
Dox: Doxorubicin
YB-1: Y-box binding protein 1
MLC-2v: Myosin light chain-2 expression
Nkx2.5: NK2 homeobox 5
GATA-4: GATA-binding protein 4
TAC: Transverse aortic constriction
Ang II: Angiotensin II
TNF-α: Tumor necrosis factor α
MYH7: Myosin heavy chain 7
AR: Androgen receptor
MAFbx: Muscle ubiquitin E3 ligases muscle atrophy F-box
BNP: Brain natriuretic peptide
ANP: Atrial natriuretic peptide
DRG: Dorsal root ganglion
NGF: Nerve growth factor
TH: Tyrosine hydroxylase
CAV3: Caveolin-3
BMSCs: Bone marrow-derived mesenchymal stem cells
EMT: Mesenchymal transition
Egr-1: Early growth response 1
MMP10: Matrix metallopeptidase 10
AP-1: Activator protein 1
YAP: Yes-associated protein
TGF-β: Transforming growth factor-β
NRCs: Neonatal rat cardiomyocytes
GADD153: Growth arrest and DNA damage-inducible protein 153
HCV: Hepatitis C virus
PKCα: Protein kinase C
HCM: Hypertrophic cardiomyopathy
DCM: Dilated cardiomyopathy
CHDs: Congenital heart defects
SMCs: Smooth muscle cells
ALS: Amyotrophic lateral sclerosis
